# Implementation of Evidence-Informed Behavioral Health Models to Improve HIV Health Outcomes for Black Men Who Have Sex With Men (Black MSM Initiative): Protocol for Program Evaluation

**DOI:** 10.2196/36025

**Published:** 2022-07-25

**Authors:** Sarah E Hodge, Ashani Johnson-Turbes, Shauna St. Clair Flemming, Meredith Passero, Melinda Tinsley, Thelma Iheanyichukwu, The Black MSM Initiative Study Group

**Affiliations:** 1 NORC at the University of Chicago Bethesda, MD United States; 2 HIV/AIDS Bureau Health Resources & Services Administration US Department of Health and Human Services Rockville, MD United States; 3 See Acknowledgments

**Keywords:** HIV infections, sexual and gender minorities, outcome assessment, health care, delivery of health care, African Americans, homosexuality, male, mental health services, HIV epidemic, minority population, epidemiology, peer support, health service, health outcomes, HIV, public health

## Abstract

**Background:**

The HIV epidemic in the United States disproportionately affects Black communities. Nearly half of Black men who have sex with men (MSM) will be diagnosed with HIV in their lifetime. There is a significant unmet need for behavioral health care services among Black MSM, and untreated behavioral health needs make it less likely the person is retained in HIV care.

**Objective:**

This paper offers a description of the Implementation of Evidence-Informed Behavioral Health Models to Improve HIV Health Outcomes for Black Men who have Sex with Men (Black MSM) Initiative, a program to integrate clinical care and behavioral health/supportive services for Black MSM with HIV. The Black MSM Initiative is funded through the Health Resources & Services Administration HIV/AIDS Bureau Ryan White HIV/AIDS Program (RWHAP) Part F Special Projects of National Significance.

**Methods:**

The components of the Black MSM Initiative include providing technical assistance to 8 Initiative demonstration sites; conducting a comprehensive and culturally responsive, mixed method, multisite evaluation; and disseminating evaluation findings and lessons learned to the RWHAP community.

**Results:**

As of December 31, 2020, demonstration sites enrolled 809 clients in the multisite evaluation. The research team will continue evaluation data collection through December 2021 for analysis and dissemination starting in 2022. The Black MSM Initiative fully supports the US Department of Health and Human Services’ Ending the HIV Epidemic in the United States Initiative.

**Conclusions:**

In order to succeed, providers and programs will need to engage populations traditionally considered “hard to reach,” like many people receiving RWHAP services. Findings and lessons learned from the Black MSM Initiative will expand the tool kit of solutions to support and retain Black MSM in HIV care, furthering the goals of the Ending the HIV Epidemic Initiative and the RWHAP.

**International Registered Report Identifier (IRRID):**

DERR1-10.2196/36025

## Introduction

### Background

The HIV epidemic in the United States disproportionately impacts Black communities, and Black men who have sex with men (MSM) bear an unequal share of that burden. Black MSM account for less than one percent of the US population, but between 20%-25% of all new US HIV infections [1]. Furthermore, if current diagnosis rates persist, about half of Black MSM in the United States will be diagnosed with HIV in their lifetime, versus 1 in 11 white MSM [2]. In comparison, lifetime risk for all men in the United States is 1 in 68 [3]. In addition, Black MSM are less likely to achieve viral suppression than the national average for clients served by the Ryan White HIV/AIDS Program (RWHAP) [4]. This is in part because of the intersecting ways in which Black MSM are marginalized and experience disparities in access to health care [5].

Black MSM with HIV may have intersectional experiences with stigma or discrimination based on race, sexual orientation, gender expression or gender identity, and/or HIV status. Perceived stigma or discrimination, along with existing medical mistrust within the community, may influence Black MSM’s ability to safely and comfortably access clinical or behavioral health care [6]. Cumulative experiences of discrimination or trauma negatively impact mental and physical health. Research indicates experiences of racism increase vulnerability to depression and other mental health conditions [7]. Unfortunately, there is significant unmet need for behavioral health care among Black MSM. Untreated behavioral health needs may also make it less likely a person is retained in HIV clinical care [8]. Further, compared to other MSM receiving outpatient HIV medical care, Black MSM face shortages of stable housing, nutritional support, substance use treatment, and mental health services [9].

Given these substantial inequities, the US Department of Health and Human Services Health Resources & Services Administration (HRSA) HIV/AIDS Bureau (HAB) RWHAP Part F Special Projects of National Significance (SPNS) funded an initiative to address the unique clinical, behavioral health, and social support needs of Black MSM with HIV. The purpose of the Implementation of Evidence-Informed Behavioral Health Models to Improve HIV Health Outcomes for Black Men who have Sex with Men (“the Black MSM Initiative”) is to engage and retain Black MSM in HIV medical care and supportive services by addressing their behavioral health needs [10]. This aim of this paper is to describe the Black MSM Initiative’s strategies to integrate clinical care, behavioral health care, and supportive services for the intended Black MSM audience; provide technical assistance to demonstration sites; design for the mixed method, culturally responsive evaluation (CRE) of the Initiative; and disseminate evaluation findings and lessons learned.

### Overview of the Black MSM Initiative

Through the Black MSM Initiative, HRSA HAB funds 8 demonstration sites to adapt and implement one of 4 evidence-informed models of care (MOCs) expected to improve linkage to care, engagement, retention, and HIV health outcomes. Demonstration sites integrate clinical and behavioral health care to serve the comprehensive needs of Black MSM with HIV. The MOCs are a youth-focused case management intervention [11], Strength Through Youth Livin’ Empowered (STYLE) [12], Brothers United/the Damien Center’s Linkage to Care program [13], and Project Silk [14]. All models were originally developed to improve HIV care and treatment and/or HIV health outcomes for youth and/or adult men of color.

In addition, to support implementation and evaluation of the demonstration sites’ interventions, HRSA HAB funds NORC at the University of Chicago (NORC) to serve as the Evaluation and Technical Assistance Provider (ETAP). The ETAP’s four goals are to (1) provide technical assistance and capacity building assistance to the demonstration sites; (2) implement a comprehensive multisite evaluation; (3) develop and disseminate successful models, findings, best practices, and lessons learned to the RWHAP community; and (4) promote successful replication of effective evidence-informed interventions and/or MOCs through trainings, publications, and other dissemination products. Together, these activities facilitate engagement and retention of Black MSM in HIV care.

The demonstration sites represent a range of organizational types and experiences, including academic medical centers, federally qualified health centers, community-based organizations, and hospital/health systems. The nonclinical demonstration sites partner with other clinics to provide HIV clinical and behavioral health care to their clients. Sites are located across 7 states and operate in both urban and suburban settings. Seven of the sites are RWHAP Parts A, B, C, D, or F Dental recipients. Table 1 provides an overview of the 8 demonstration sites.

Table 2 provides an overview of the 4 MOCs and adaptations made to each MOC to better fit the site context or local population.

**Table 1:** Overview of the demonstration sites.

Demonstration site	City, state	Selected model of care	Organizational type	Length of intervention	Target population
Christian Community Health Center	Chicago, IL	Youth-focused case management intervention	Community-based clinic (federally qualified health center)	12 months	Black MSM^a^ LWH^b^ aged ≥18 years
Parkland HIV Services Department, Dallas County Hospital District	Dallas, TX	Youth-focused case management intervention	Hospital system	9 months	Black MSM LWH aged 17-34 years
Duke University	Durham, NC	Strength Through Youth Livin’ Empowered (STYLE)	Academic program, nonclinical	12 months	Black MSM LWH aged 18-35 years
Friends Research Institute, Inc.	Los Angeles, CA	Youth-focused case management intervention	Community research site	3 months	Black MSM LWH aged 18-65 years
GMHC Inc	New York, NY	Project Silk	Community-based organization	12 months	Black MSM LWH aged 18-45 years
CrescentCare	New Orleans, LA	Brothers United/The Damien Center’s Linkage to Care program	Community-based clinic (AIDS service organization, federally qualified health center)	12 months	Black MSM and Black transgender men LWH aged ≥13 years
East Bay Advanced Care, Sutter Bay Hospitals	Oakland, CA	Youth-focused case management intervention	Hospital system	6-18 months, depending on enrollment date	Black MSM LWH (no age range)
Project ARK at Washington University	St. Louis, MO	Youth-focused case management intervention	Academic program, clinical	6 months	Black MSM LWH aged 18-29 years

^a^MSM: men who have sex with men.

^b^LWH: living with HIV.

**Table 2:** Models of care and site adaptations.

Model of care	Number of sites	Model summary	Core components	Site adaptations
Youth-focused case management intervention	5	The goal of this intervention was to improve retention in HIV care for young Latino and African American MSM. Case managers provided supportive services to fill participants’ identified needs for housing, nutrition support, substance abuse treatment, or mental health services.	Two bachelor-level case managers Clinic- and venue-based outreach 24-month intervention Psychosocial case management services	Expanding eligible age range Shortening intervention length (between 3 and 18 months) Using peer case managers
Strength Through Youth Livin’ Empowered (STYLE)	1	Strength Through Youth Livin’ Empowered (STYLE) was designed to improve retention in HIV care through a social marketing campaign, outreach to youth and provision of HIV testing services, and a coordinated medical and social support network for recently diagnosed and lost-to-care youth with HIV.	Medical case manager and peer outreach worker Targeted venue-based and social marketing outreach 24-month intervention Case management and ancillary support services	Combining case manager and outreach worker position Adding a behavioral health provider Shortening intervention length to 12 months Using an app to engage clients virtually
Project Silk	1	Project Silk was a youth-led, adult-supported drop-in program for LGBTQ^a^ individuals that offered recreation opportunities, food and snacks, health services like HIV/sexually transmitted infection testing, access to mental health counseling, and community resources.	Engagement with House and Ball community Recreation-based safe space focusing on artistic expression Colocated supportive services	Expanding the target population to include non–House and Ball clients
Brothers United/the Damien Center’s Linkage to Care program	1	This program, run by an Indianapolis community-based organization, provided comprehensive wraparound and supportive services to the Black LGBTQ community. The program offered prevention and testing. Those who tested positive were referred to services that help them engage in care.	Linkage to Care specialists One-stop shop for comprehensive care and referral services Support groups	Adding an on-site behavioral health therapist

^a^LGBTQ: lesbian, gay, bisexual, transgender, queer.

### Target Population

The Black MSM Initiative target population is Black MSM with HIV. Specifically, to be eligible to participate, a client must be HIV positive; aged 13 and older; identify as a Black man who has sex with men (including cisgender men, transgender men, and gender nonconforming individuals assigned male at birth); and fit into one of the following categories: newly diagnosed/new to care, never entered into care, fallen out of care, at risk of falling out of care, or not virally suppressed. Risk factors for falling out of care are operationalized as ongoing behavioral health issues (eg, mental health and/or substance use disorders), a history of irregular engagement in care, housing and/or employment instability, a history of sexually transmitted infections, or a history of negative experiences in a health care setting. Demonstration sites could impose more restrictive eligibility criteria as needed for their intervention (eg, must have access to a smartphone).

The Black MSM Initiative uses several key definitions to describe project activities. As defined by HRSA, *HIV care services* include all HIV care and treatment services allowable through the RWHAP; *behavioral health* refers to mental or emotional well-being and/or actions that affect wellness; and *behavioral health care* includes screening and treatment for substance use disorders, alcohol and drug addiction, and serious psychological distress, suicide, and mental disorders [15]. *Evidence-informed interventions* are strategies, models, or approaches that have proven effective or shown promise as a methodology, practice, or means of improving the care and treatment of people with HIV.

### Purpose

This paper describes the rationale for integrating clinical care, behavioral health care, and supportive services, and presents the Black MSM Initiative model, which includes providing technical assistance to demonstration sites, conducting a culturally responsive multisite evaluation, and disseminating evaluation findings and lessons learned.

## Methods

### Strategies for Integrating Behavioral Health

The primary objective of the Black MSM Initiative is to integrate clinical and behavioral health care to improve health outcomes. Demonstration sites designed a variety of intervention activities to accomplish this goal, including peer support groups, individual counseling, motivational interviewing during case management sessions, and SMS text messaging services that screen for behavioral health concerns. To provide behavioral health care to enrolled clients, 6 demonstration sites offer in-house behavioral health services and all demonstration sites offer referrals to off-site behavioral health and supportive services, including substance use treatment.

To integrate clinical and behavioral health care, demonstration sites employ multiple strategies that include holding regular case conferences with case management staff, behavioral health staff, and HIV clinical providers to discuss clients and coordinate care; meeting regularly to identify and address client needs; improving cross-care team communication; and engaging behavioral health staff as members of the intervention team.

Additionally, to integrate care, demonstration sites follow clients through their receipt of behavioral health care and endeavor to eliminate barriers to care (eg, offering transportation to provider visits). Some demonstration sites provide warm handoffs from case managers/peer navigators to behavioral health providers and have providers introduce themselves during clinical or case management appointments. Case management/peer navigation staff aim to ensure clients understand behavioral health care and work to normalize this care. For example, some demonstration sites offer social support groups facilitated by peer staff, which discuss behavioral health needs, reframe mental or behavioral health as mental wellness, and focus on overall well-being. In addition to integrating behavioral health care, these techniques are valuable for sustaining client engagement.

### Provision of Technical Assistance

The ETAP provides technical assistance (TA) and capacity building assistance (CBA) to the demonstration sites in intervention adaptation, implementation, and evaluation. The ETAP assesses the needs of the demonstration sites and coordinates trainings, provides resources, and develops tools. In addition to responding to specific requests from the demonstration sites, TA/CBA is provided proactively through regular teleconferences with each demonstration site, office hours, multisite meetings, site visits, teleconferences with frontline staff, and website resources. To leverage demonstration sites’ expertise, the ETAP encourages and facilitates peer learning and development through these venues. Areas of TA/CBA include marketing and recruitment; retention; local evaluation; data collection and submission for the multisite evaluation; dissemination; sustainability; staff training, support, and self-care; and COVID-19 adaptations. To identify demonstration sites’ needs and inform the provision of proactive TA, the ETAP conducted needs assessments, held calls with the sites, and developed a site typology.

The *needs assessments* gathered information on current capacity and anticipated needs for support around program adaptation, staffing, recruitment, implementation, retention, Institutional Review Board approval, local evaluation, and sustainability planning. The ETAP used findings from the needs assessments to identify gaps in site capacity and tailor TA provision. *Initial calls* with the sites facilitated in-depth learning about each sites’ local context and MOC adaptations, implementation and local evaluation plans, and anticipated challenges and TA needs. Information from the calls was used to address the immediate needs of sites and identify topics for future cross-site discussions. These calls also informed the development of the multisite evaluation. The ETAP continues to meet with the demonstration sites monthly to monitor recruitment, enrollment, and evaluation progress and discuss TA needs. Following conduct of the first needs assessment and initial calls, the ETAP developed a *typology* to document and compare the demonstration sites, their selected MOCs, MOC adaptations, local evaluation design, and capacity for contribution to the multisite evaluation. As the ETAP learns more about each site, the ETAP updates the typology to facilitate meaningful provision of TA/CBA.

### Multisite Evaluation Strategy

To assess the impact of the Black MSM Initiative on expected outcomes, the ETAP is conducting a culturally responsive, sequential, transformative, mixed method evaluation. The purpose of the multisite evaluation (MSE) is to (1) assess processes associated with implementing evidence-informed interventions, including barriers and facilitators to implementation (*process study*); (2) assess whether the evidence-informed interventions impact clinical and behavioral health outcomes (*outcomes study*); and (3) assess the costs of adapting and implementing the interventions by measuring labor and programmatic expenditures (*cost analysis*). The evaluation uses document review, demonstration site calls, site visits, key informant interviews, a pre-post client survey, demonstration site capture of implementation data, and client-level outcome data. Demonstration sites gather data at baseline and, at minimum, 6 and 12 months postbaseline. Table 3 summarizes the 3 evaluation components and how they align with MSE methods.

**Figure 1:**
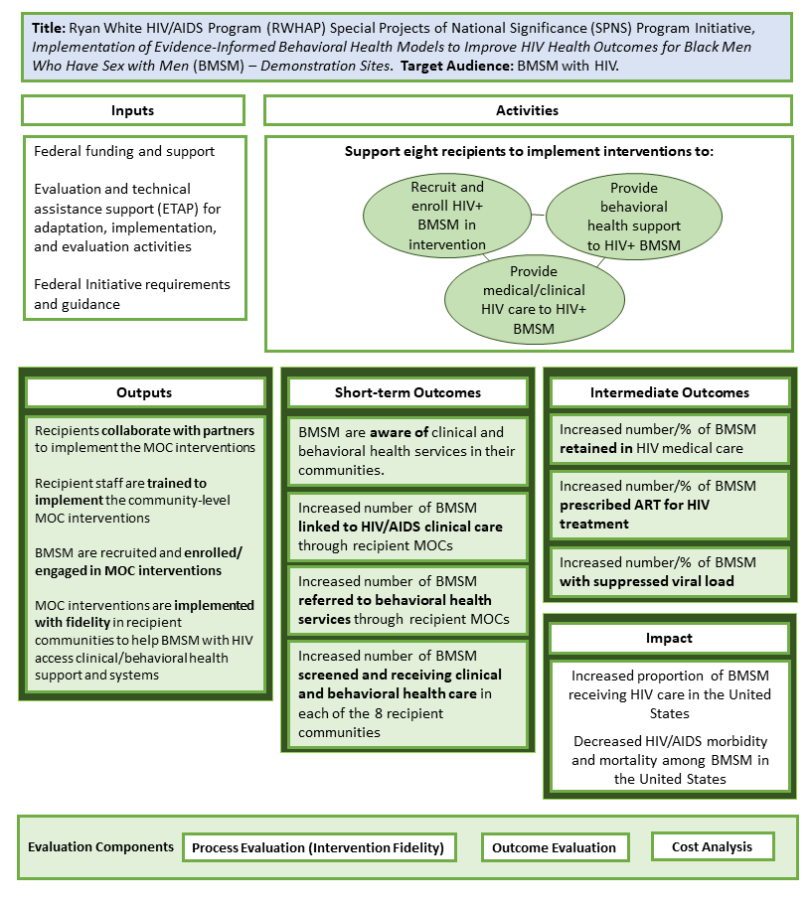
Black MSM Initiative logic model. ART: antiretroviral treatment; BMSM: Black men who have sex with men; MOC: model of care; MSM: men who have sex with men.

To design the evaluation, the ETAP first created an Initiative logic model that depicts the Black MSM Initiative’s inputs, activities, outputs, and expected outcomes (Figure 1).

**Table 3:** Overview of the evaluation studies, aims, and methods.

Evaluation component	Study aim	Method type	Data collection frequency
Process study	Aim 1: Assess processes associated with implementing evidence-informed interventions, including barriers and facilitators to implementation.	Qualitative, quantitative	Baseline in year 1, twice in year 2, twice in year 3
Outcome study	Aim 2: Assess whether evidence-informed interventions impact clinical/behavioral health outcomes.	Quantitative	Baseline in year 1, twice in year 2, twice in year 3
Cost analysis	Aim 3: Assess the costs of adapting and implementing the interventions/MOCs by measuring labor and programmatic costs and expenditures incurred by each site.	Qualitative, quantitative	Once in year 1, once in year 2, once in year 3

Following development of the logic model, the ETAP, in consultation with RWHAP SPNS Project Officers and demonstration site project teams, identified specific process study, outcome study, and cost analysis evaluation questions. The process study questions explore what factors influence adaptation and implementation of interventions; which intervention components are delivered with fidelity; and how clients are recruited to participate. Outcome study questions include whether the Initiative contributed to changed awareness of HIV care and behavioral health care/supportive services; linkage to care, screening, referral to care, receipt of care, retention, and engagement outcomes; and antiretroviral therapy (ART) prescription and HIV viral load outcomes. The cost analysis examines costs per client served and costs per key programmatic outcome. After determining the evaluation questions, the ETAP identified the appropriate evaluation framework and design.

The MSE uses a CRE approach [16,17]. The ETAP proposed CRE because it focuses on recognizing the centrality of culture, and invites and legitimizes the diverse perspectives of demonstration sites, stakeholders, and Black MSM with HIV. CRE seeks to enhance the social, political, and economic conditions of persons from traditionally underrepresented and underserved communities by executing valid evaluations [18]. In practice, this means the ETAP designed study questions and instruments to explore and account for the role of community context, demographics, socioeconomics, sexuality, gender, politics, and culture.

**Figure 2:**
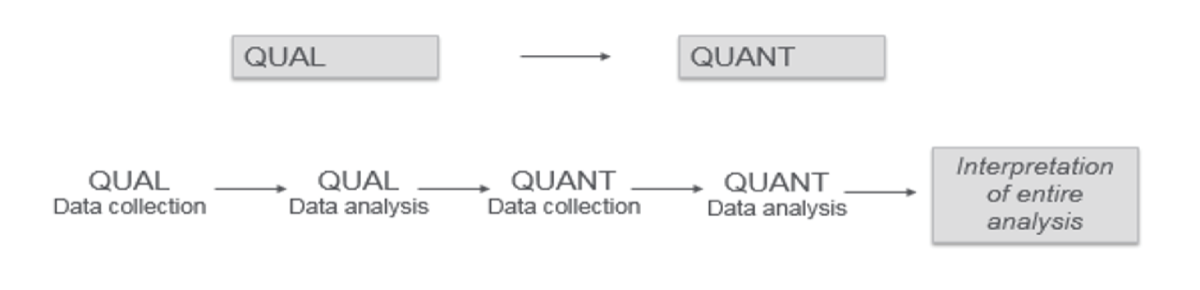
Design of the multisite evaluation. QUAL: qualitative; QUANT: quantitative.

The ETAP uses a sequential, transformative strategy to collect and analyze relevant process and outcome measures [19]. Transformative research [20,21] focuses on studying the lives and experiences of diverse, often-marginalized groups; requires collaborative inquiry so as not to marginalize clients; and advances an agenda to improve clients’ lives [22]. This evaluation design allows, at minimum, two distinct, sequential data collection phases (either qualitative or quantitative) and a theoretical perspective to guide the evaluation (Figure 2) [19,23].

Qualitative MSE data collection instruments include a document review extraction form to systematically extract relevant data from site-developed documents; protocols for annual site visits; and tailored, semistructured interview guides for annual key informant interviews. Quantitative instruments include a patient survey administered at baseline and 6 and 12 months postbaseline; a Demonstration Site Assessment Tool to track intervention service encounters; a Main Outcomes Instrument to collect individual-level clinical outcomes data also at baseline and 6 and 12 months postbaseline; and an Interview-Assisted Cost Worksheet to collect site-level cost information.

To analyze qualitative data, the ETAP uses applied thematic analysis to identify common themes, patterns, and interrelationships in the data relevant to expected outcomes and answer linked evaluation questions. To analyze quantitative data, the ETAP aggregates data across all demonstration sites to analyze and answer linked evaluation questions. Using generalized linear mixed effects models, the ETAP compares client outcomes between baseline and each of the follow-up time points (6 and 12 months) and assesses clustering effects within demonstration sites. The ETAP performs stratified analysis to determine whether changes in outcomes vary by subgroup. To complete the cost analysis, the ETAP uses micro-costing methodology to collect data on labor, supplies, facilities, and other cost inputs. The ETAP combines different cost inputs to create estimates of the aggregated costs of each intervention and total costs per client served, and will use bootstrapping if needed to estimate uncertainty. As the final step, the ETAP mixes the quantitative and qualitative data to ensure the findings are complementary and answer overarching MSE study questions.

### Ethics Approval

Ethical conduct of the study was overseen by the NORC Institutional Review Board, which determined the study to not be human subjects research because the ETAP did not receive any identifying information about participants. Identifiers were stored locally on secure servers; the ETAP audited data security during site visits. Several demonstration sites also obtained Certificates of Confidentiality to further protect participant privacy. All study procedures were conducted in accordance with the ethical standards set forth in the 1975 Declaration of Helsinki.

### Dissemination Planning

The ETAP supports the dissemination of best practices and lessons learned from the Initiative and promotes replication of successful interventions. The ETAP facilitates timely dissemination of results and encourages cross-site collaboration through a Publications & Dissemination Committee, which includes representatives from HRSA, NORC, and each demonstration site. Complementary to dissemination, the ETAP collaborates with the RWHAP National Coordinating Resource Center, regional RWHAP AIDS Education and Training Centers, and other organizations to promote replication across the RWHAP community.

The ETAP collaborates with the demonstration sites to develop a range of forthcoming dissemination and replication products, including implementation manuals, implementation toolkits, monographs, and spotlights. These products will provide background information, implementation guidance, and best practices for organizations interested in replicating efforts of the demonstration sites. Future publications will describe MSE methods and results, as well as notable adaptations, challenges/successes, and lessons learned, especially related to integrating behavioral health care and supporting clients amid COVID-19 and other 2020 social unrest. The demonstration sites will share their experiences with implementing the adapted MOCs and findings from local evaluations in future dissemination products.

### Impact of the COVID-19 Public Health Emergency

In the United States, the COVID-19 Public Health Emergency (PHE) exacerbates persistent systemic inequities. According to the Centers for Disease Control and Prevention, interrelated social determinants of health like income, education, occupation, and access to health care put racial and ethnic minorities at increased risk of COVID-19–related morbidity and mortality [24]. Early evidence shows disparities in COVID-19 cases and deaths among people of color across the United States [25]; the rate of COVID-19 cases among Black or African American non-Hispanic persons is 2.6 times greater than the rate among White non-Hispanic persons [26]. In addition, the Centers for Disease Control and Prevention identifies immunocompromised individuals, including people with HIV, as being at increased risk for poor COVID-19 outcomes [27].

Following declaration of the COVID-19 PHE, stay-at-home orders, and social distancing guidelines, most demonstration sites closed their doors and ceased recruitment to mitigate viral spread. They continued to engage with existing clients via phone, text, or video meetings to the extent possible. In addition, demonstration sites reported an increase in basic needs among enrolled clients during the COVID-19 pandemic. Many clients continue to experience significant hardship due to the COVID-19 pandemic including job loss, housing instability, mental health concerns, and distress due to being sick or having friends and/or family members who are sick. By July 2020, all demonstration sites resumed some intervention activities and reopened for in-person or virtual engagement, depending on state, local, and institutional guidelines. However, most client interactions remain virtual, including evaluation data collection. Virtual engagement has required many demonstration sites to design new case management protocols and obtain Institutional Review Board or institutional approvals for the use of tools like Zoom videoconferencing software.

## Results

The Black MSM Initiative continues to collect and synthesize robust evidence to examine proposed solutions to engage and retain Black MSM in HIV care. In response to the needs assessment, the ETAP offered TA and CBA on intervention adaptation, implementation, and evaluation. These efforts included developing guidance and definitions for the target audience and sample size; providing recommendations for recruiting eligible clients; developing and identifying measures for local evaluations; and supporting online platforms for sites with limited data collection infrastructure. Ongoing opportunities for TA include supporting retention and project engagement, boosting data and survey completion rates, and facilitating development of dissemination products that are cohesive, visually appealing, and accessible to a variety of audiences.

Sites continue to integrate behavioral health services through group, peer, and one-on-one support; facilitate relationships between behavioral health and clinical care teams; and reduce identified barriers to care. Teams have also contended with challenges related to the COVID-19 PHE as services moved into virtual spaces and clients experienced significant hardships related to job loss and housing instability. In particular, the COVID-19 PHE has made it difficult for some clients to sustain engagement in care when other survival needs took precedence. In response, sites have seen an uptick in social services needs and continue to work with community partners and clients to ensure those needs are met.

As of December 31, 2020, demonstration sites enrolled 809 clients in the MSE. Data collection will continue through December 2021.

## Discussion

The study team began sharing early results from the MSE in December 2019 and expects to submit final results for publication in Spring 2022. In order to facilitate future replication by RWHAP providers and other organizations, the demonstration sites will create publicly available Implementation Manuals and Toolkits. NORC will also disseminate evaluation findings via future publications. As the Black MSM Initiative continues, the ETAP will increasingly support the sites with dissemination, replication, and sustainability activities.

The Black MSM Initiative fully supports the US Department of Health and Human Services’ Ending the HIV Epidemic in the United States Initiative, which aims to reduce the number of new HIV infections by at least 90% in the next 10 years. In order to succeed, providers and programs will need to engage populations traditionally considered “hard to reach,” like many people receiving RWHAP services. Findings and lessons learned from the Black MSM Initiative will expand the toolkit of solutions to support and retain Black MSM in HIV care, furthering the goals of the Ending the HIV Epidemic Initiative and the RWHAP.

## Abbreviations

ART: antiretroviral treatment
CBA: capacity building assistance
CRE: culturally responsive evaluation
ETAP: Evaluation and Technical Assistance Provider
HAB: HIV/AIDS Bureau
HRSA: Health Resources & Services Administration
MOC: model of care
MSE: multisite evaluation
MSM: men who have sex with men
NORC: NORC at the University of Chicago
PHE: public health emergency
RWHAP: Ryan White HIV/AIDS Program
SPNS: Special Projects of National Significance
STYLE: Strength Through Youth Livin’ Empowered
TA: technical assistance
